# Supplementary material for: Quantitative analysis of protein-protein interactions and post-translational modifications in rare immune populations
Source: Nat Commun. 2017 Nov 15;8:1524. doi: 10.1038/s41467-017-01808-6 (PMC5688095; doi:10.1038/s41467-017-01808-6)
Supplement: Supplementary file 1 — Supplementary Information [file 41467_2017_1808_MOESM1_ESM.pdf]

a

Aire + Sirt1 PLA

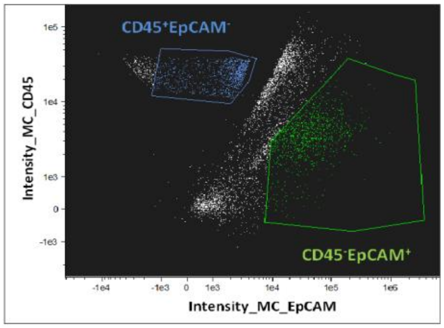

Intensity_MC_EPCAM, Intensity_MC_CD45

Population	Count	%Gated	Mean	Median	Std. Dev.
All	4893	100	18880.35, 14957.43	6904.07, 10171.72	41316.11, 15769.14
CD45- EpCAM+	938	19.2	62165.14, 3998.58	39168.85, 3515.73	73423.77, 2664.35
CD45+	997	20.4	1546.24, 23610.99	1830.14, 23153.37	1372.43, 7131.8

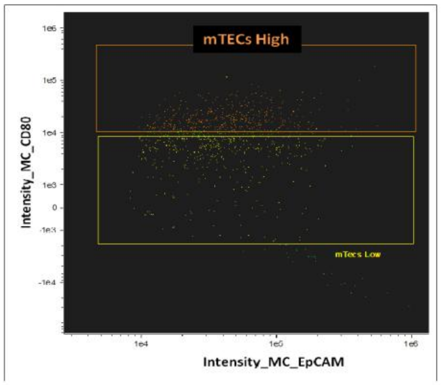

Intensity_MC_EPCAM, Intensity_MC_CD80

Population	Count	%Gated	Mean	Median	Std. Dev.
CD45- EpCAM+	938	100	62165.14, 12621.57	39168.85, 9652.7	73423.77, 14180.65
mTecs Low & CD45- EpCAM+	403	43	53675.37, 4086.54	35631.5, 4362.14	50967.09, 2682.16
mTecs High & CD45- EpCAM+	426	45.4	59831.79, 22554.28	42508.69, 17525.15	63205.71, 15442.28

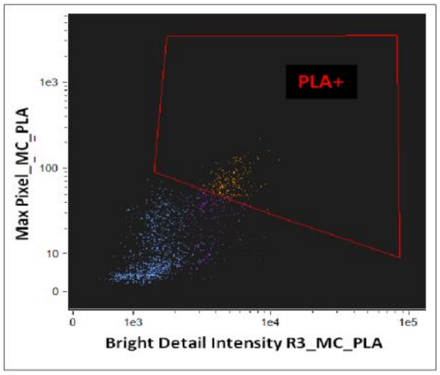

Bright Detail Intensity R3_MC_PLA, Max Pixel_MC_PLA

Population	Count	% Gated	Mean	Median	Std. Dev.
mTecs High & CD45- EpCAM+	426	100	4299.7, 46.35	4114, 42.04	1569.26, 31.83
PLA+ & mTecs High & CD45- E...	168	39.4	5274.12, 76.4	5019, 69.07	1324.2, 27.21
CD45+	997	100	1473.79, 12.81	1426, 7.807	500.06, 11.16
PLA+ & CD45+	0	0	NaN, NaN	NaN, NaN	NaN, NaN

Mouse MINUS and Rabbit PLUS Probes only

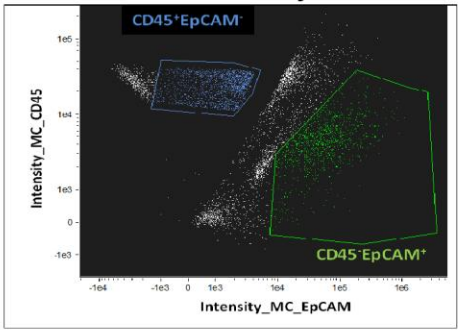

Intensity_MC_EPCAM, Intensity_MC_CD45

Population	Count	% Gated	Mean	Median	Std. Dev.
All	4856	100	23850.25, 17668.49	4144.59, 15319.18	61449.33, 16393.56
CD45- EpCAM+	991	20.4	84820.33, 5157.54	50125.96, 4216.41	110748.49, 3968.62
CD45+	1466	30.2	1368.39, 24526.19	1409.05, 24143.3	1209.53, 7963.58

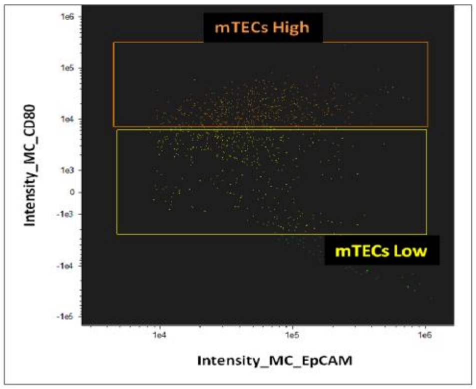

Intensity_MC_EPCAM, Intensity_MC_CD80

Population	Count	%Gated	Mean	Median	Std. Dev.
CD45- EpCAM+	991	100	84820.33, 10828.38	50125.96, 7214.76	110748.49, 16169.43
mTecs Low & CD45- EpCAM+	399	40.3	60067.97, 2153.1	41362.06, 2286.03	59941.13, 2211.21
mTecs High & CD45- EpCAM+	487	49.1	81217.36, 20687.12	53699.35, 16004.73	89113.25, 17641.17

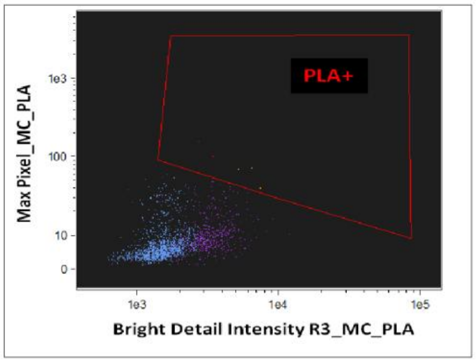

Bright Detail Intensity R3_MC_PLA, Max Pixel_MC_PLA

Population	Count	%Gated	Mean	Median	Std. Dev.
mTecs High & CD45- EpCAM+	487	100	3464.6, 12.12	3258, 9.404	1158.09, 10.9
PLA+ & mTecs High & CD45- E...	4	0.82	5463.75, 82.8	5858.5, 70.05	2029.15, 47.75
CD45+	1466	100	1554.27, 7.513	1497.5, 5.612	440.56, 6.86
PLA+ & CD45+	1	0.07	3449, 101.27	3449, 101.27	0, 0

Supplementary Figure 1

PLIC analysis of Aire and Sirt1 interaction in mTECs

(a) Gating strategy for PLIC positive signal of Aire+Sirt1 interaction compared to probes only sample. Representative dot-plot graphs of WT and probes only samples of Aire+Sirt1 PLIC analysis (using the IDEAS 6.2 ImageStream software). Initial gating was done for CD45^{+}Epcam^{-} (CD45) and Epcam^{+}CD45^{-} (TECs) populations (upper panel); sub-gating for mTEC^{hi} (CD80^{high}) and mTEC^{lo} (CD80^{low}) populations (middle panel) and for PLIC^{+} signal (PLA+, lower panel).

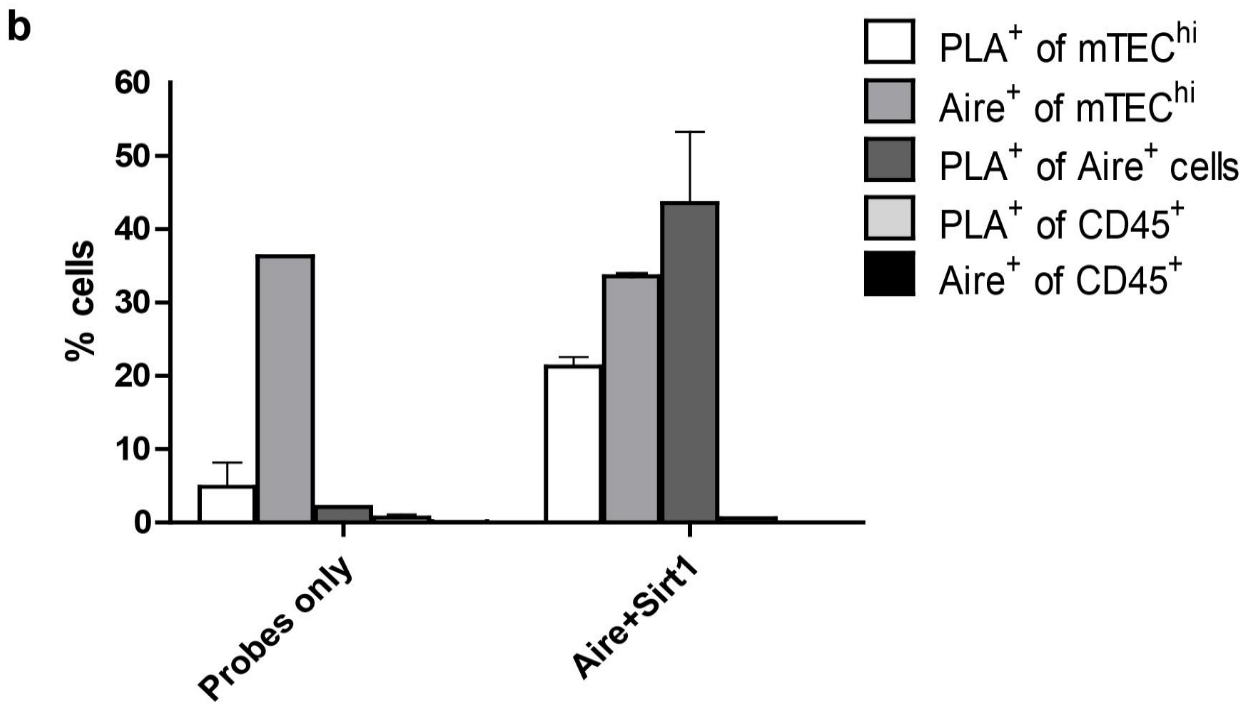

Supplementary Figure 1

PLIC analysis of Aire and Sirt1 interaction in mTECs

(b) Quantitative analysis of Aire and Sirt1 interaction in the different sub populations, presented as percentage of positive cells for – PLA in mTEC^{hi}, Aire in mTEC^{hi}, PLA in Aire^{+} (of mTEC^{hi}), PLA in CD45^{+} and Aire in CD45^{+} cells, isolated from WT mice compared to probes background (Average of 2 independent experiments, n=2, s.e.m).

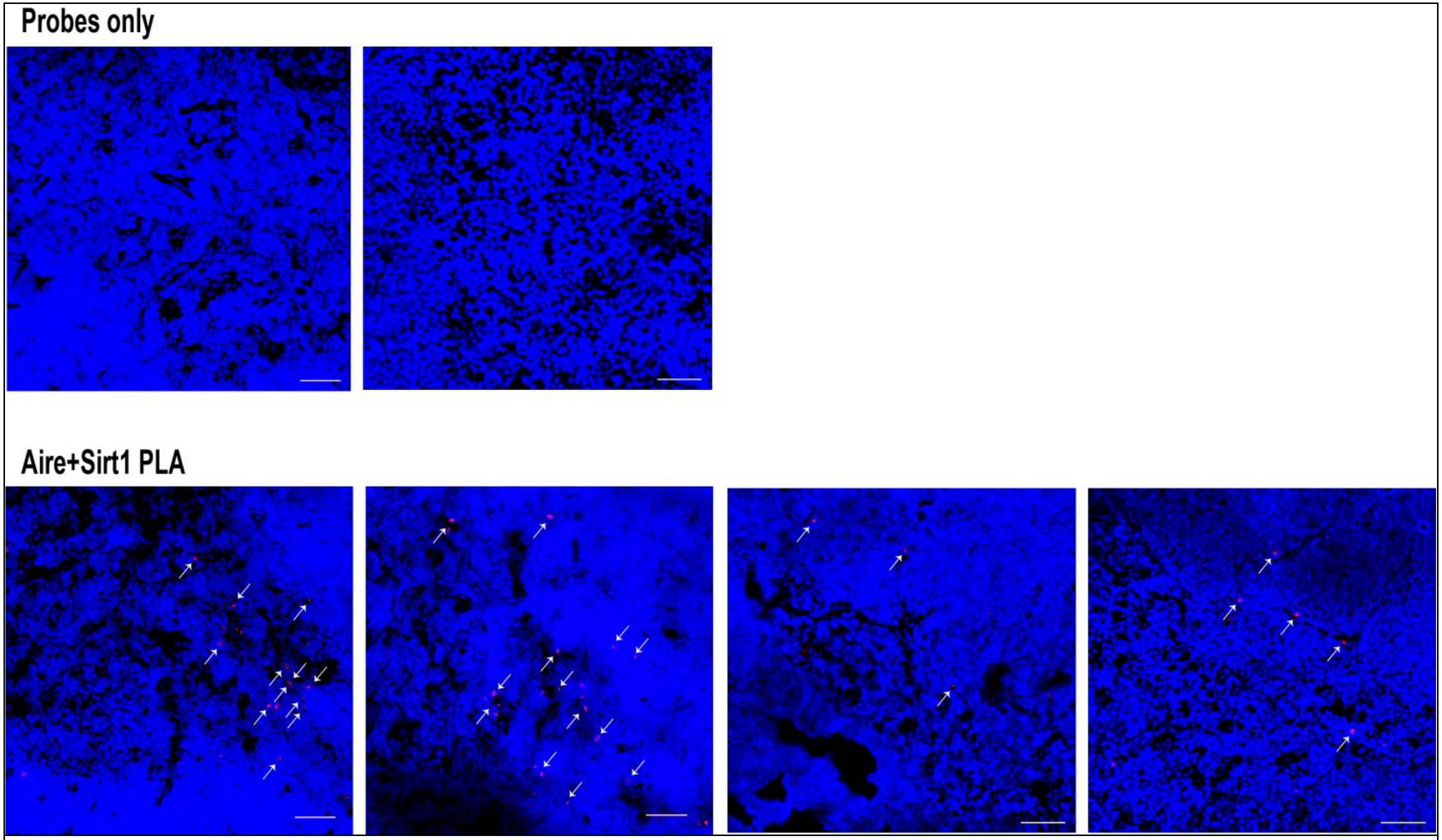

Supplementary Figure 2

Aire and Sirt1 interaction detected by standard PLA and confocal microscopy

PLA probing Aire and Sirt1 interaction in WT thymic sections analyzed by confocal microscopy. The upper panel shows negative controls (NC), in which only PLA probes with no primary antibodies were used; the lower panel shows staining of Aire and Sirt1. Protein interaction appears as red dots in the medullary region (highlighted by white arrows). Nuclei were stained with DAPI. Scale bar 50μM.

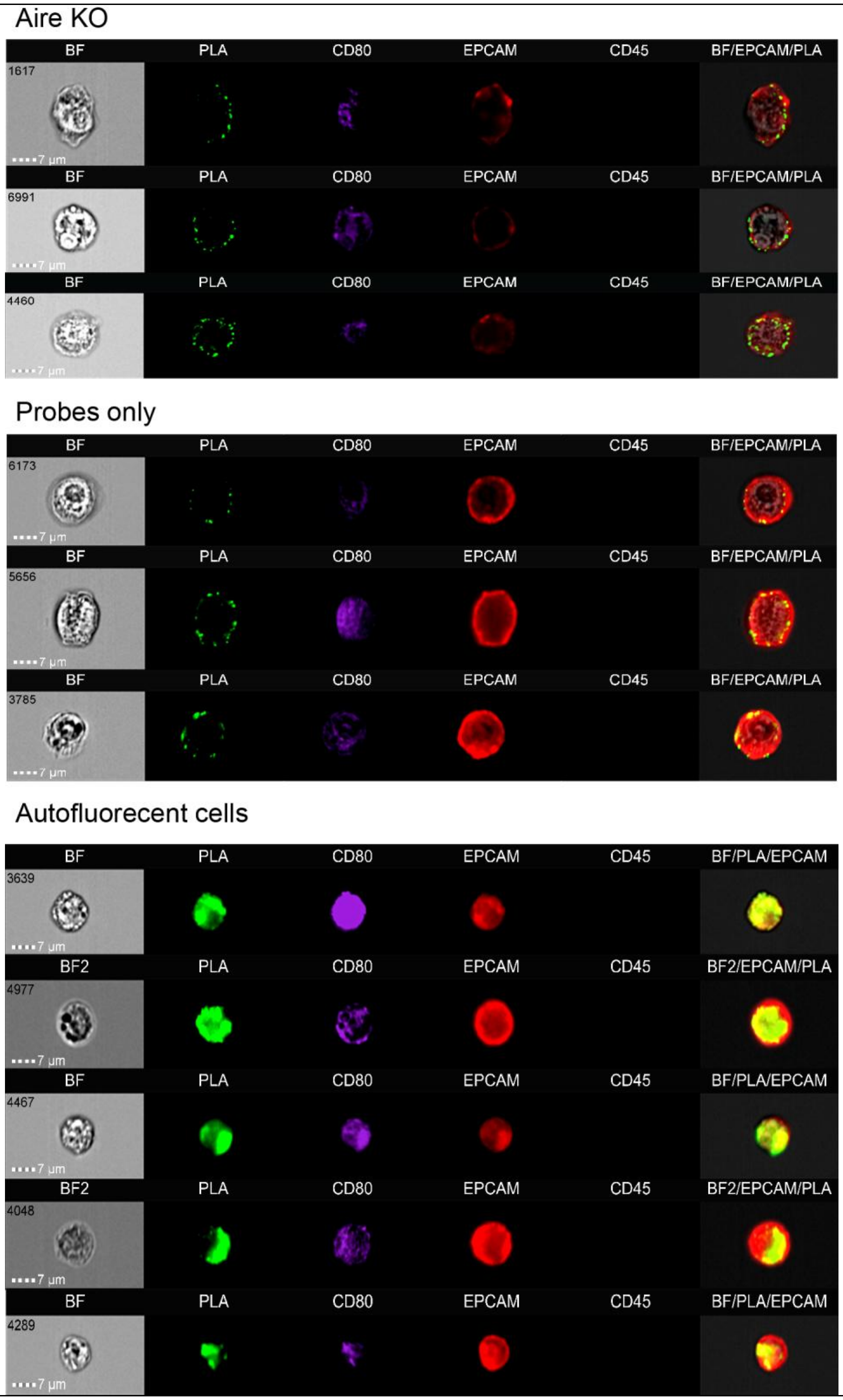

Supplementary Figure 3

PLA false-positive signal caused by non-specific binding or cells autofluoerscence

Representative images of PLA false-positive signal caused by probes non-specific membranal binding and cells auoflourescence in mTEC^{hi} isolated from *Aire*$^{-/-}$ mice, or probes only sample (WT), prior to a corrected analysis gating. Images are presented as (left to right): bright field (BF), PLA (green), staining of CD80 (PB), EpCAM (APC), CD45 (APC/Cy7) and BF/PLA/EpCAM overlay (merged). Scale bar 7μM.

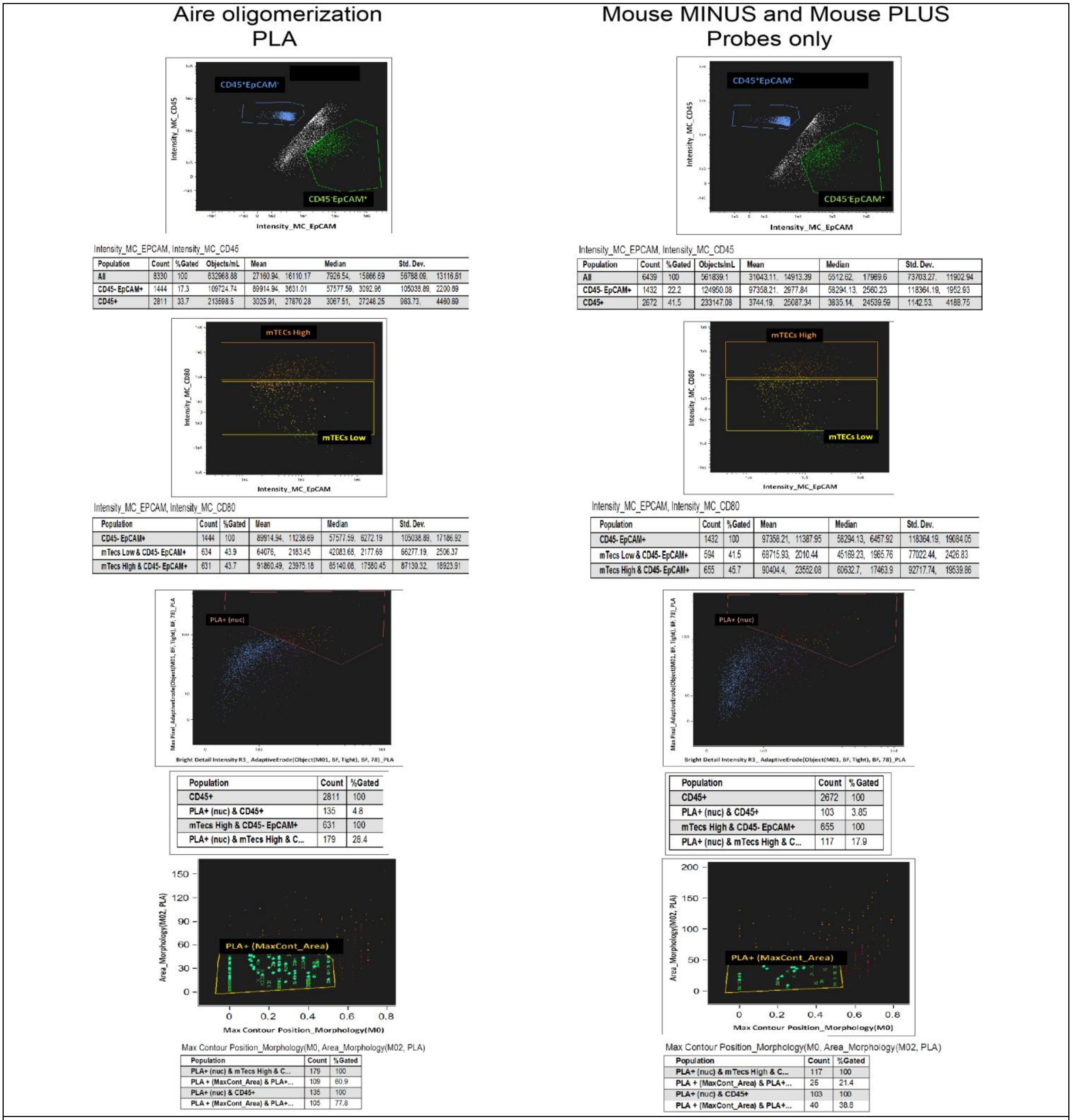

Aire oligomerization PLA

Intensity_MC_EPCAM, Intensity_MC_CD45

Population	Count	%Gated	Objects/mL	Mean	Median	Std. Dev.
All	8330	100	632968.88	27160.94, 16110.17	7926.54, 15866.69	56788.09, 13116.81
CD45- EpCAM+	1444	17.3	109724.74	89914.94, 3631.01	57577.59, 3092.96	105038.89, 2200.69
CD45+	2811	33.7	213598.5	3025.91, 27870.28	3067.51, 27248.25	963.73, 4460.69

Intensity_MC_EPCAM, Intensity_MC_CD80

Population	Count	%Gated	Mean	Median	Std. Dev.
CD45- EpCAM+	1444	100	89914.94, 11238.69	57577.59, 6272.19	105038.89, 17186.92
mTecs Low & CD45- EpCAM+	634	43.9	64076, 2183.45	42083.68, 2177.69	66277.19, 2506.37
mTecs High & CD45- EpCAM+	631	43.7	91860.49, 23975.18	65140.06, 17580.45	87130.32, 18923.91

Population	Count	%Gated
CD45+	2811	100
PLA+ (nuc) & CD45+	135	4.8
mTecs High & CD45- EpCAM+	631	100
PLA+ (nuc) & mTecs High & C...	179	28.4

Max Contour Position_Morphology(M0, Area_Morphology(M02, PLA)

Population	Count	%Gated
PLA+ (nuc) & mTecs High & C...	179	100
PLA + (MaxCont_Area) & PLA+...	109	60.9
PLA+ (nuc) & CD45+	135	100
PLA + (MaxCont_Area) & PLA+...	105	77.8

Mouse MINUS and Mouse PLUS Probes only

Intensity_MC_EPCAM, Intensity_MC_CD45

Population	Count	%Gated	Objects/mL	Mean	Median	Std. Dev.
All	6439	100	561839.1	31043.11, 14913.39	5512.62, 17989.6	73703.27, 11902.94
CD45- EpCAM+	1432	22.2	124950.08	97358.21, 2977.84	58294.13, 2560.23	118364.19, 1952.93
CD45+	2672	41.5	233147.08	3744.19, 25087.34	3835.14, 24539.59	1142.53, 4188.75

Intensity_MC_EPCAM, Intensity_MC_CD80

Population	Count	%Gated	Mean	Median	Std. Dev.
CD45- EpCAM+	1432	100	97358.21, 11387.95	58294.13, 6457.92	118364.19, 19084.05
mTecs Low & CD45- EpCAM+	594	41.5	68715.93, 2010.44	45169.23, 1965.76	77022.44, 2426.83
mTecs High & CD45- EpCAM+	655	45.7	90404.4, 23552.08	60632.7, 17463.9	92717.74, 19539.86

Population	Count	%Gated
CD45+	2672	100
PLA+ (nuc) & CD45+	103	3.85
mTecs High & CD45- EpCAM+	655	100
PLA+ (nuc) & mTecs High & C...	117	17.9

Max Contour Position_Morphology(M0, Area_Morphology(M02, PLA)

Population	Count	%Gated
PLA+ (nuc) & mTecs High & C...	117	100
PLA + (MaxCont_Area) & PLA+...	25	21.4
PLA+ (nuc) & CD45+	103	100
PLA + (MaxCont_Area) & PLA+...	40	38.8

Supplementary Figure 4

Gating strategy for PLIC positive signal of Aire oligomerization compared to probes only sample

Representative dot-plot graphs of WT and probes only samples of Aire oligomerization PLIC analysis (using the IDEAS 6.2 ImageStream software). Initial gating was done for $CD45^{+}Epcam^{-}$ (CD45) and $Epcam^{+}CD45^{-}$ (TECs) populations (first panel) then sub-gating for $mTEC^{hi}$ ($CD80^{high}$) and $mTEC^{lo}$ ($CD80^{low}$) populations (second panel). CD45 and $mTEC^{hi}$ populations were further gated for $PLIC^{+}$ signal (PLA+ (nuc), third panel). Further gating was done according to signal localization (nuclear) in order to reduce the percentage of false positive interactions detected (PLA+(MaxCont_Area), fourth panel).

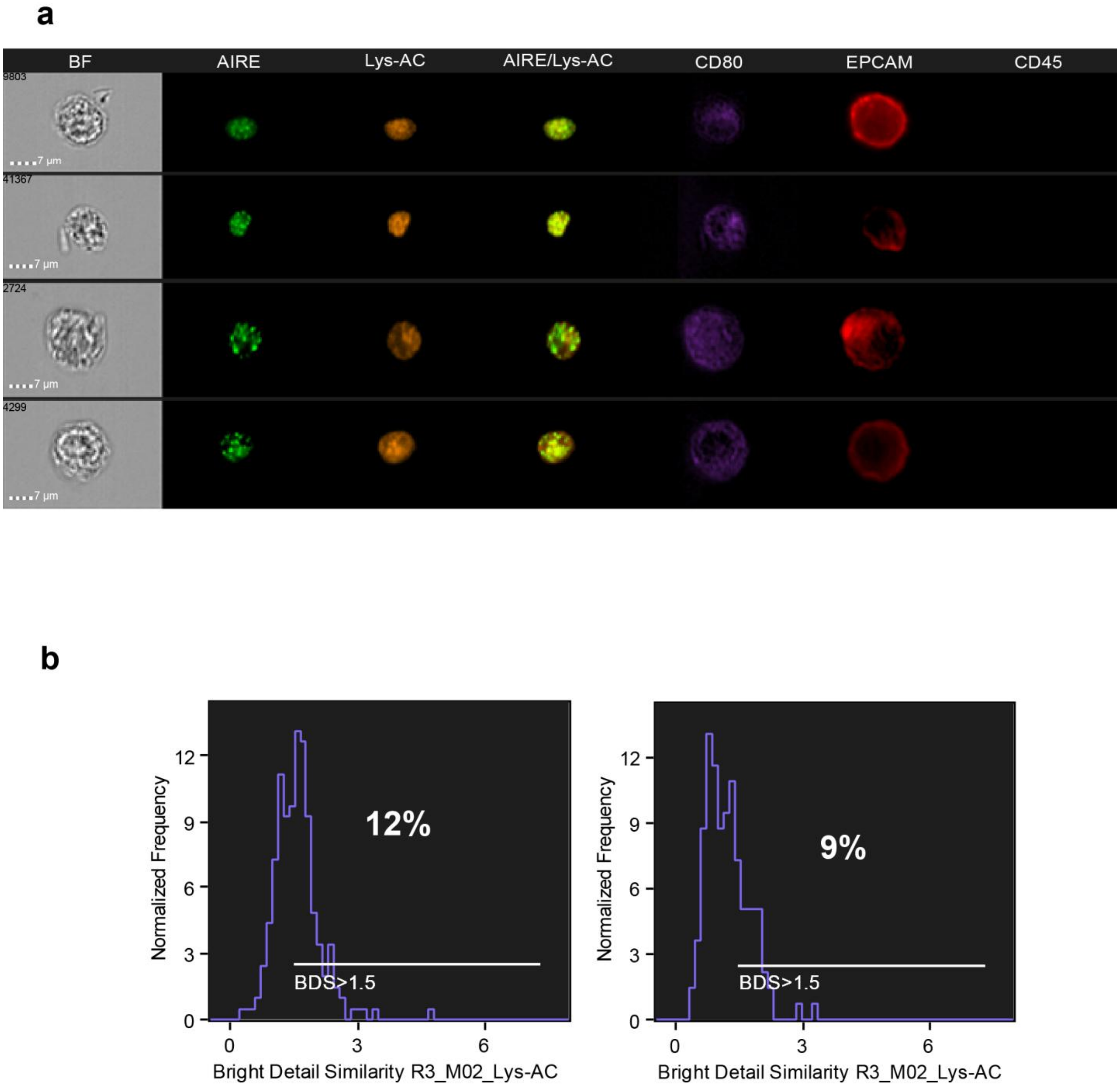

Supplementary Figure 5

Co-localization analysis of Aire and lysine acetylation in mTECs

(a) Representative images of Aire and lysine-acetylation staining in $mTEC^{hi}$, presented as (left to right): bright field (BF), staining of Aire (FITC), lysine-acetylation (AF555), Aire/lysine-acetylation overlay (merged), CD80 (PB), EpCAM (APC) and CD45 (APC/Cy7). Scale bar 7μM. (b) Imaging flow cytometry analyzing the co-localization of Aire and lysine acetylation in $mTEC^{hi}$ based on bright detail similarity (BDS) feature (see methods for detailed explanation). Positive co-localization is considered as BDS>1.5 (2 independent experiments, n=1).
